# A novel lncRNA BADLNCR1 inhibits bovine adipogenesis by repressing *GLRX5* expression

**DOI:** 10.1111/jcmm.15181

**Published:** 2020-05-25

**Authors:** Hanfang Cai, Mingxun Li, Wang Jian, Chengchuang Song, Yongzhen Huang, Xianyong Lan, Chuzhao Lei, Hong Chen

**Affiliations:** ^1^ Key Laboratory of Animal Genetics, Breeding and Reproduction of Shaanxi Province College of Animal Science and Technology Northwest A&F University Yangling Shaanxi China; ^2^ College of Animal Science and Technology Yangzhou University Yangzhou Jiangsu China

**Keywords:** adipogenesis, BADLNCR1, bovine, *GLRX5*, *KLF2*

## Abstract

Adipogenesis is a complex cellular process, which needs a series of molecular events, including long non‐coding RNA (lncRNA). In the present study, a novel lncRNA named BADLNCR1 was identified as a regulator during bovine adipocyte differentiation, which plays an inhibitory role in lipid droplet formation and adipogenic marker gene expression. CHIPR‐seq data demonstrated a potential competitive binding motif between BADLNCR1 and sterol regulatory element‐binding proteins 1 and 2 (SREBP1/2). Dual‐luciferase reporter assay indicated target relationship between KLF2 and BADLNCR1*.* Moreover, after the induction of KLF2, the expression of adipogenic gene reduced, while the expression of BADLNCR1 increased. Real‐time quantitative PCR (qPCR) showed that BADLNCR1 negatively regulated mRNA expression of *GLRX5* gene, a stimulator of genes that promoted formation of lipid droplets and expression of adipogenic genes. GLRX5 could partially reverse the effect of BADLNCR1 in bovine adipocyte differentiation. Dual‐luciferase reporter assay stated that BADLNCR1 significantly reduced the enhancement of C/EBPα on promoter activity of *GLRX5* gene. Furthermore, CHIP‐PCR and CHIRP‐PCR confirmed the suppressing effect of BADLNCR1 on binding of C/EBPα to GLRX5 promoter. Collectively, this study revealed the molecular mechanisms underlying the negative regulation of BADLNCR1 in bovine adipogenic differentiation.

## 1 INTRODUCTION

Adipose tissue is an extremely important and complex part for animals and humans. In addition to providing a protective layer for the organs and keeping body from the cold, it is also an organ that restores energy and works as an endocrine tissue which has a profound impact on the metabolism of other tissues, the regulation of appetite, insulin sensitivity, immunological responses and vascular disease.^1^, ^2^ Additionally, for beef cattle, the amount and distribution of fat significantly influence the carcass and meat quality.^3^, ^4^, ^5^ Consequently, breeding for good carcass adipose is an important research in the development of beef industry.

Formation of adipose tissue is called adipogenesis, which is controlled by transcriptional activities of peroxisome proliferator‐activated receptor γ (PPARγ) that cooperates with CCAAT/enhancer‐binding proteins (C/EBPs) to stimulate the expression of adipogenic genes, such as adipocyte fatty acid‐binding protein/adipocyte P2 (FABP4/AP2) and fatty acid translocase (FAT/CD36) that give rise to adipocyte phenotype.^5^, ^6^ Except for coding genes, lncRNA is a set of non‐coding RNA considered to be longer than 200 nt with little or no coding potential.^6^ Recently, a plenty of lncRNA has been identified to be involved in diverse biological processes, including genomic imprinting,^7^ chromatin modification,^8^ cancer metastasis,^9^, ^10^ neurogenesis,^11^ myogenic development^12^ and adipogenesis.^13^, ^14^ Li et al^15^ discovered hundreds of lncRNA that differently expressed in mature adipocytes compared to pre‐adipocytes of cattle. However, the detailed function of only one lncRNA was revealed. The roles of most lncRNA in bovine adipogenesis are unknown.

Glutaredoxin 5 *(GLRX5)* is a 156 amino acid mitochondrial protein, which is evolutionarily conserved.^16^ It is necessary for iron‐sulphur clusters transfer, which is required for normal iron homoeostasis.^17^ Also, *GLRX5* is involved in protein lipoylation, since mutation in *GLRX5* gene could impair transfer of [Fe‐S] to lipoate synthase enzyme.^18^ As a family member of *GLRX5*, GLRX1 was elevated 70% in the adipose tissue of the obese and 45% fat calorie‐fed rats.^19^ Additionally, there is a great difference in mitochondrial proteome between the different adipose tissues.^20^ But it is still unclear how GLRX5 participates in adipogenesis.

Given this, in this study, the negative role of a novel lncRNA, bovine adipocyte differentiation–related long non‐coding RNA 1 (BADLNCR1) in bovine adipogenic differentiation, is revealed. Its genome‐wide binding is analysed to reveal its regulation mechanisms. As its neighbour, *GLRX5* is found to be its target, which positively regulates bovine adipocyte differentiation. Finally, why BADLNCR1 represses *GLRX5* transcription is investigated. These data provide a novel insight into lncRNA and *GLRX5* in molecular regulation of bovine adipogenesis.

## 2 MATERIALS AND METHODS

All experiments were approved by the Review Committee for the Use of Animal Subjects of Northwest A&F University. All experiments were performed in accordance with relevant guidelines and regulations.

### 2.1 Cell culture and Oil Red staining

Bovine primary pre‐adipocytes were isolated from inguinal fat of newborn calf using type I collagenase digestion as previously described.^21^, ^22^ Pre‐adipocytes were maintained in Dulbecco's modified Eagle's medium (DMEM) supplemented with 10% foetal bovine serum (FBS) (Gibco, 26 140 079), 100 μg/mL streptomycin and 100 U/mL penicillin. After reaching 100% confluence, pre‐adipocyte was induced by differentiation medium containing 10% FBS, 100 μg/mL streptomycin, 100 U/mL penicillin, 10 µg/mL insulin (Sigma, I6634), 0.5 mmol/L 3‐isobutyl‐1‐methylxanthine (IBMX) (Sigma, I5879) and 1 µmol/L dexamethasone (Sigma, D4902) for 2‐3 days, and then incubated with DMEM containing 10% FBS, 100 μg/mL streptomycin, 100 U/mL penicillin and 10 µg/mL insulin. Culture medium was changed every two days. 293A cell was maintained in DMEM supplemented with 10% FBS, 100 μg/mL streptomycin and 100 U/mL penicillin.

For Oil Red O staining, cells were washed with PBS for three times and fixed with 4% paraformaldehyde for 10 minutes. After that, cells were washed twice with deionized water and then stained with Oil Red O solution (0.3% Oil Red O, 60% isopropanol and 40% PBS) for 20 minutes. Before imaging, cells were washed with PBS for four times. All the procedure was performed at room temperature.

### 2.2 Rapid amplification of cDNA ends (RACE)

In order to gain the full‐length sequence of BADLNCR1, SMARTer RACE cDNA Amplification Kit (Clotech, 634859) was used to perform 5’RACE according to the instructions. And 3’RACE was performed referring to Frohman Scotto‐Lavino.^23^ The template RNA was extracted from adult adipose tissue of cattle. The gene‐specific primer used for 5’ RACE was 5’‐GATTACGCCAAGCTTCTGCCAGTTTTCTCTTCCTGTCGG‐3’, and two gene‐specific primers used for 3’RACE were inner: 5’‐TGCCATGTGCAATTTTCC‐3’ and outer: 5’‐AGAGGAAGCTGAGGCATG‐3’.

### 2.3 Plasmid, nucleotide and cell transfection

To construct the expression vectors, the full length of BADLNCR1, *GLRX5*, *KLF2* and CEBPα was amplified and constructed into pcDNA3.1(+) vector. To translate BADLNCR1 in vitro, the fragment of BADLNCR1 was cloned into pET‐28a vector. Also, the genomic fragments containing upstream 2,000 bp of transcriptional start site of BADLNCR1 and *GLRX5* gene were constructed into pGL3‐basic vector to construct luciferase reporter plasmids, respectively. Primers used in plasmid construction are listed in Table S3.

The siRNAs specifically target on BADLNCR1, *GLRX5* and negative control siRNA were ordered from GenePharma. The sequences of siRNAs for BADLNCR1 were 5’GUGUGUGCACUUCGAUUAUTT‐3’, for *GLRX5* were 5’‐UCCCGCAAGUGUACCUUAATT‐3’, and for negative control were UUCUCCGAACGUGUCACGUTT‐3’. All the transfection was performed using Lipofectamine 2000 (Invitrogen, 11668027) according to the instruction.

### 2.4 Cell fractionation and fluorescent in situ hybridization (FISH)

Cytoplasmic and nuclear RNA of pre‐adipocytes and mature adipocytes were extracted using PARIS Kit (Invitrogen, AM1921) based on its instruction. Cy3‐labelled BADLNCR1 probes for FISH assay were purchased from RiboBio. And RNA FISH was carried out using FISH Kit (RiboBio, lnc1100285) following its instruction.

### 2.5 CHIRP‐seq, RNA pull‐down and mass spectrum, and CHIP

In order to obtain genome‐wide binding of BADLNCR1, CHIRP assays with both odd and even probes (Table S4) that ordered from RiboBio were performed referring to Chu et al^24^ using bovine mature adipocyte. The final eluted DNA was sequenced, and initial bioinformatics analysis was done in Novogene.

RNA pull‐down assay was performed according to Klattenhoff et al^25^ using bovine mature adipocyte. The eluted protein was sent to Saicheng (Guangzhou) for mass spectrum. According to Zhou et al,^26^ CHIP was carried out. Antibody anti‐rabbit CEBPα (cat No. PA5‐77911) was purchased from Invitrogen. Primers used in CHIP‐PCR and CHIRP‐PCR to evaluate the enhancement of *GLRX5* promoter were forward primer: 5’‐AGAATGGAGAAAGCGGTGGG‐3’ and reverse primer: 5’‐GCGAGAAGTCCAGTGAGACC‐3’.

### 2.6 Bioinformatics analysis

Secondary structure analysis was performed using Vienna RNAfold server (http://rna.tbi.univie.ac.at/cgi‐bin/RNAfold.cgi), to do minimum free energy structure analysis. Coding potential score was analysed by coding potential calculator (CPC) (http://cpc.cbi.pku.edu.cn/). The putative transcription factor‐binding sites were predicted using the following online tools: MatInspector (http://www.genomatix.de/), JASPAR database (http://hfaistos.uio.no:8000/) ^27^ and Promo (http://alggen.lsi.upc.es/cgi‐bin/promo_v3/promo/promoinit.cgi?dirDB=TF_8.3). ^28^, ^29^ GO (http://www.geneontology.org/) annotation was evaluated by the DAVID software.^30^, ^31^

### 2.7 Real‐time quantitative PCR (qPCR)

Total RNA from cells was extracted using TRIzol kit (Takara, 9108). cDNA was synthesized as template in qPCR using PrimeScript RT Reagent Kit (Perfect Real Time) (Takara, RR037A). Glyceraldehyde‐phosphate dehydrogenase (GAPDH) gene was chosen as internal control. The primers used are shown in Table S5. PCR was carried out in a CFX96TM Real‐Time Detection System with SYBR premix ExTaq II (TaKaRa, RR82LR). All samples were measured in triplicate. The relative expression ratios were calculated with the following formula 2^−ΔΔ^ *^C^* ^t^ as Schmittgen & Livak described.^32^

### 2.8 Luciferase assays

Cells were seeded into 96‐well plate. 36‐48 hours after transfection, the luciferase activities were measured using the Dual‐Luciferase Reporter Assay System (Promega, E1910). Luciferase activities were normalized against Renilla luciferase activity. And the experiments were repeated for three times.

### 2.9 Statistical analysis

Data were analysed by Student's *t* test using SPSS software (version 20). The results were presented as mean ± SE (Standard Error), and *P* value < .05 was considered statistically significant.

## 3 RESULTS

### 3.1 Identification of BADLNCR1

**Figure 1:**
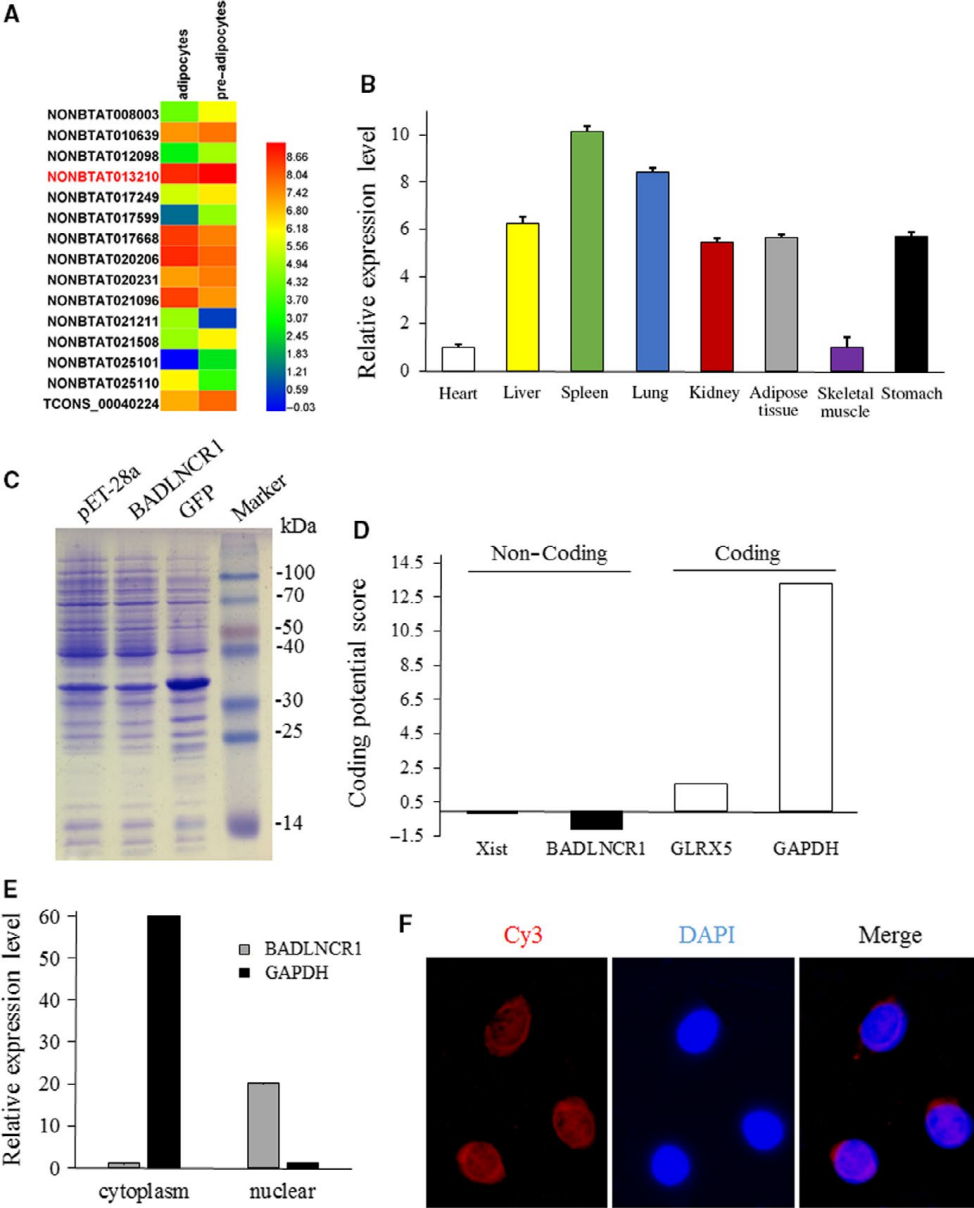
Identification of BADLNCR1 as a candidate lncRNA that is related to bovine adipogenesis. A, Heatmap of 15 differently expressed genes selected from RNA‐seq data (*P* < .01 and FDR < 0.05). B, Expression pattern of BADLNCR1 in different tissues of adult cattle. Data are presented as Mean ± SE. C, In vitro translation assay using GFP and BADLNCR1 constructs. Shown is Coomassie Blue staining. D, Coding potential of BADLNCR1 predicted by CPC. D, Expression level of BADLNCR1 in nuclear and cytoplasm detected by qPCR. Mean ± SE. D, Location of BADLNCR1 in bovine pre‐adipocytes detected by FISH. Red is BADLNCR1. Blue is cell nucleus

Previous RNA‐seq data have discovered differently expressed lncRNAs when compared bovine pre‐adipocytes to mature adipocytes.^15^ qPCR was applied to identify their expression. A novel lncRNA, NONBAT013210, had higher expression level than other lncRNAs and showed significantly decreasing expression in adipocytes (Figure 1A). Also, tissue expression pattern indicated that it highly expressed in adipose tissue of cattle (Figure 1B). Other lncRNAs showed low expression level in adipose tissue (data was not shown). So NONBAT013210 was chosen as candidate lncRNA and named as BADLNCR1.

RACE revealed that BADLNCR1 transcript was polyadenylated and reversely transcribed from chromosome 21 with two exons of 1029 bp in length (Figure S1A). A BANLNCR1 expression vector failed to produce a protein by translation assay in vitro (Figure 1C). Moreover, CPC analysis indicated that compared to protein‐coding genes, GLRX5 and GAPDH, and a famous lncRNAs, Xist, BADLNCR1 had a lower probability than all of them (Figure 1D). Secondary structure of BADLNCR1 was shown in Figure S1B.

qPCR analysis of fractionated nuclear and cytoplasmic RNA stated that BADLNCR1 was primarily expressed in nuclear; as a control, *GAPDH* gene was mainly detected in cytoplasmic (Figure 1E). This result was confirmed by FISH (Figure 1F), which also indicated that BADLNCR1 was mostly localized in nuclear.

### 3.2 BADLNCR1 inhibit bovine adipogenic differentiation

**Figure 2:**
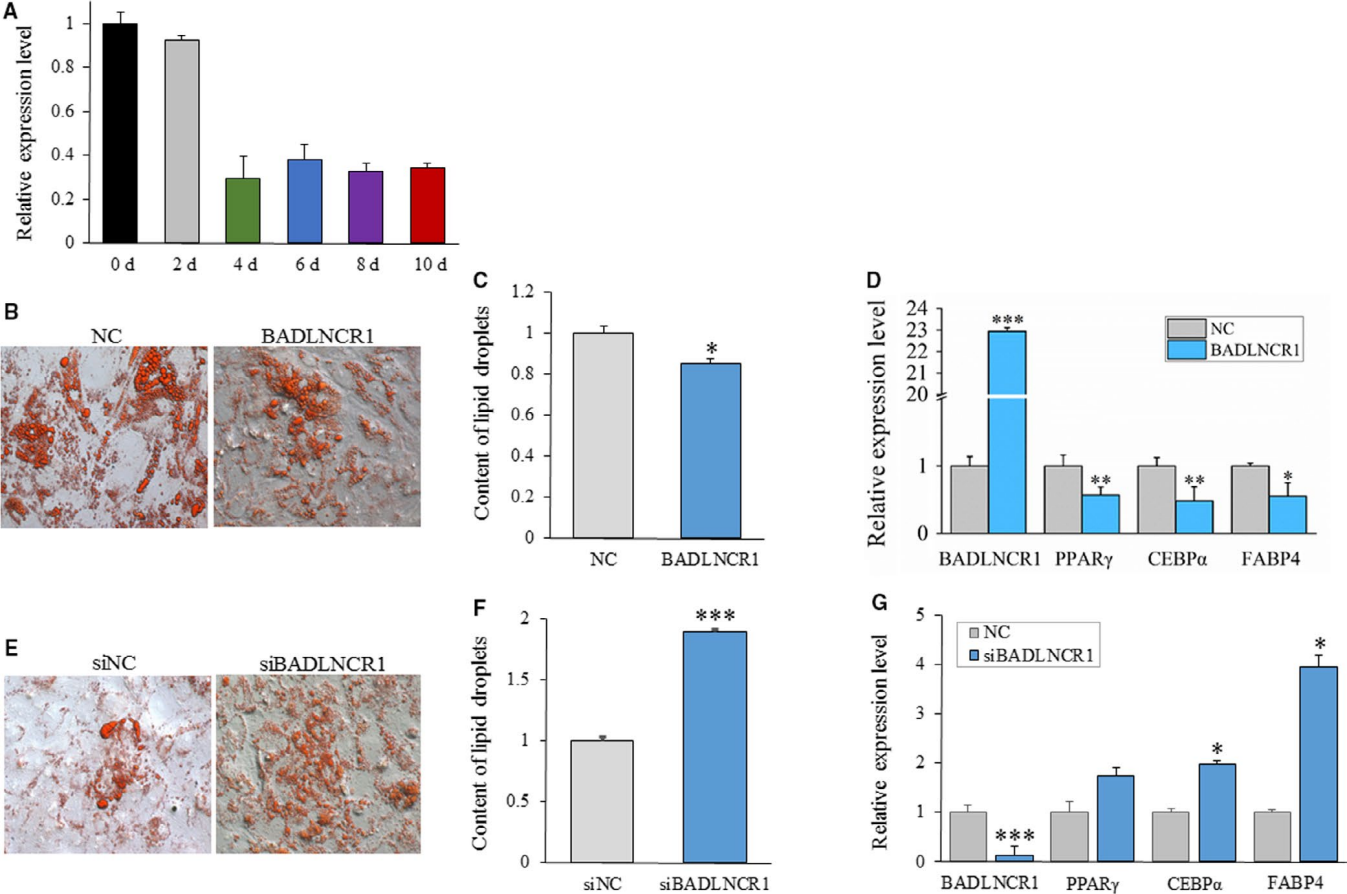
BADLNCR1 represses bovine adipogenic differentiation. A, Expression dynamics of BADLNCR1 during adipocyte differentiation. B, Red Oil Staining after overexpression of BADLNCR1. Magnification is 4000. C, Quantification of lipid droplets after overexpression of BADLNCR1 by ImageJ. D, Overexpression of BADLNCR1 inhibits mRNA expression of PPARγ, CEBPα, and FABP4. E, Red Oil staining of lipid droplets after BADLNCR1 knocked down. Magnification is 400. F, Quantification of lipid droplets after BADLNCR1 knocked down by ImageJ. G, Knock‐down of BADLNCR1 promotes expression of PPARγ, CEBPα, and FABP4.. Data are presented as Mean ± SE. *P < .05, **P < .01, ***P < .001

After adipogenic induction, BADLNCR1 displayed an obviously decreasing expression trend after 4d (Figure 2A), which indicated that BADLNCR1 may be related to final adipogenic differentiation in cattle. To explore the potential role of BADLNCR1 in bovine adipocyte differentiation, gain‐of‐function experiment was firstly carried out by transfection of pcDNA3.1(+)‐BADLNCR1 with transfection of pcDNA3.1(+) as control. Cells at 6d post‐induction were collected; adipogenic gene expression and lipid droplet formation were detected. After overexpression of BADLNCR1, significantly less lipid droplets were formed (*P* < .05) (Figure 2B,C), and the expression levels of PPARγ, CEBPα and FABP4 were significantly repressed (*P* < .05) (Figure 2B). Additionally, loss‐of‐function experiment was performed using siRNA to knock down BADLNCR1 expression in adipocytes. After BADLNCR1 was knocked down, Oil Red O staining indicated that more lipid droplets were formed (*P* < .001) (Figure 2E,F), and the expression levels of CEBPα and FABP4 raised significantly (*P* < .05), while the expression of PPARγ was moderately increased with 70% (Figure 2G). Taken together, BADLNCR1 plays as a suppressor of genes that regulates bovine adipogenic differentiation.

Transcriptional factor prediction indicated that multiple adipogenic transcriptional factors had potential binding sites on BADLNCR1 promoter, for example PPARγ, AP‐2 and SP1. Among them, *KLF2*, a negative regulator of adipogenic differentiation, has three binding sites on BADLNCR1 promoter, −1807 bp, −247 bp and −1738 bp. Dual‐luciferase activity assay and expression analysis were performed to determine whether *KLF2* regulates the transcription and expression of BADLNCR1. The promoter activity of BADLNCR1 was also increased significantly after the overexpression of *KLF2* (*P* < .01). If the binding site “GGGT” was replaced by “TTTC” (Figure S2A), *KLF2* could not change the promoter activity of BADLNCR1 significantly (Figure S2B). In addition, after the overexpression of *KLF2* in adipocytes, the mRNA level of *KLF2* increased approximately 58‐fold (*P* < .001) along with significantly decreasing expression of PPARγ (*P* < .01), CEBPα (*P* < .05) and FABP4 (*P* < .01), which confirmed the negative regulation of *KLF2* in adipogenic differentiation. Meanwhile, BADLNCR1 mRNA showed a significant increasing tendency and the expression of *GLRX5* indicated a corresponding significant reduction (Figure S2C). Collectively, these data demonstrated that *KLF2* could regulate the transcriptional activity and expression of BADLNCR1.

### 3.3 Genome‐wide binding of BADLNCR1

**Figure 3:**
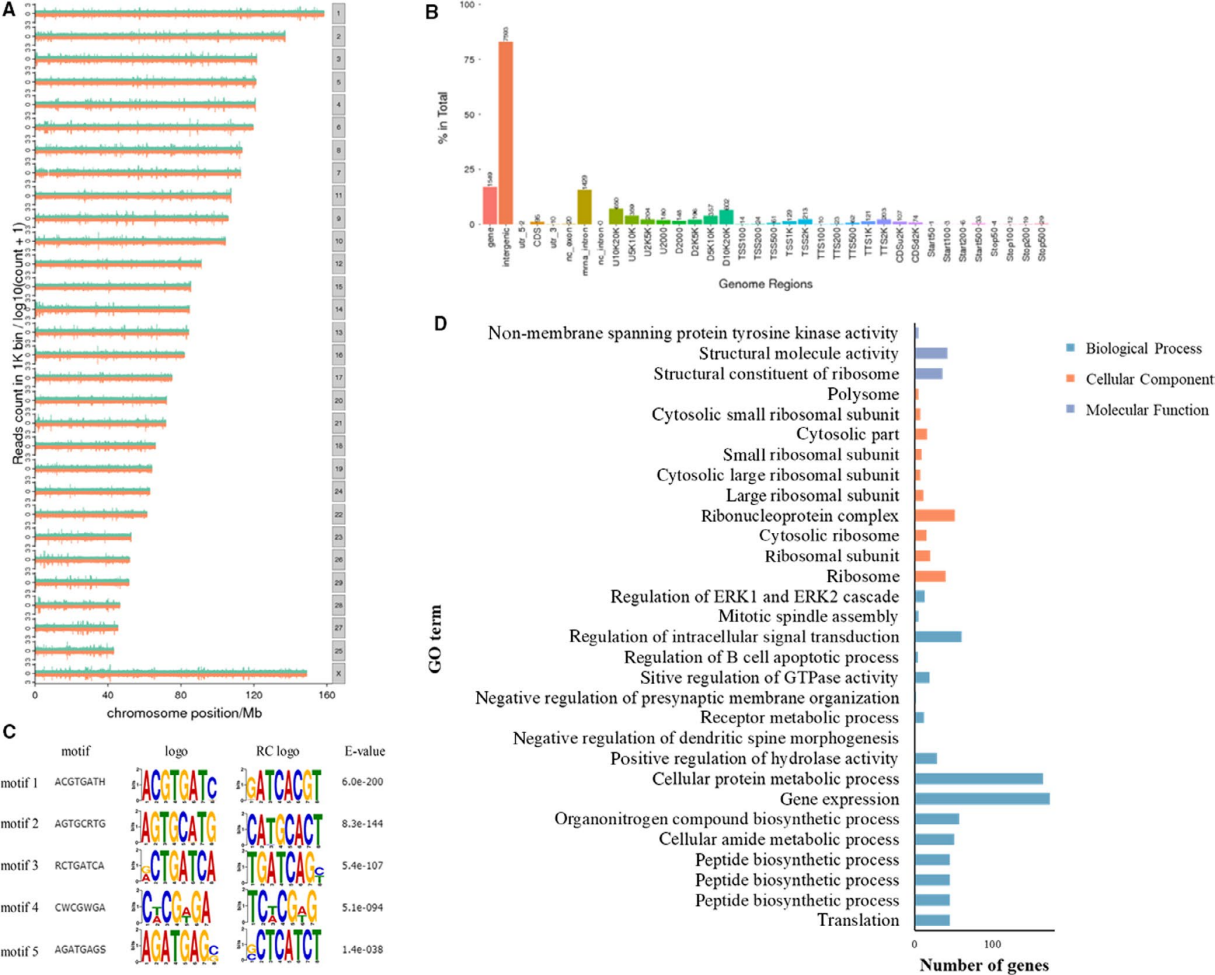
Genome‐wide binding of BADNCR1. A, Chromosome distribution of BADLNCR1‐binding peaks. B, Distribution of BADLNCR1‐binding peaks in gene functional regions. C, Top 5 significantly annotated DNA binding motif of BADLNCR1‐binding peaks. D, GO analysis of genes mapped by BADLNCR1‐binding peaks

As previous reports described, lncRNAs modulate transcription of its target genes that locates closed to the lncRNA transcription site (*cis*‐regulation) or elsewhere in different chromosomes (*trans* regulation).^33^, ^34^, ^35^ Hence, CHIRP‐seq, a method enables insights into RNA‐chromatin interactions,^24^, ^36^ was performed to reveal the potential genome‐wide DNA‐binding sites of BADLNCR1. A total of 3339 narrow peaks were obtained, and only 75 of them were mapped in the same chromosome 21 as BADLNCR1. Major of these peaks were mapped in different chromosomes (Figure 3A). So BADLNCR1 acts both in *cis* and in *trans*. By genome‐wide mapping, most peaks (80%) were located in intergenic region. In terms of the distance to genes, more peaks were located at ~10 kb downstream or upstream from genes (Figure 3B), which is the gene expression regulation region.^37^ This indicated the potential role of BADLNCR1 in regulation of gene expression. Using DMEM motif analysis, top five significantly enriched DNA‐binding motifs of BADLNCR1, ACGTGATH, AGTGCRTG, RCTGATCA, CWCGWGA and AGATGAGS were found (Figure 3C). Interestingly, after comparing them to known motif using JASPAR analysis, motif 1 and motif 2 highly resemble binding sequence for sterol regulatory element‐binding protein 1 (SREBP1) and SREBR2 (Table S1), which are two important transcriptional factors that bind on promoter of adipogenic genes to promote their expression, such as PPARγ, and promote adipocyte differentiation.^38^, ^39^, ^40^ Based on this, it supposed that BADLNCR1 could have an effect on adipocyte differentiation by impacting the biding of SREBP1/SREBP2 on expression of adipogenic genes.

Gene Ontology (GO) terms’ enrichment (http://www.geneontology.org/) of genes to which those peaks mapped was analysed. In total, 583 GO terms were significantly enriched (*P* < .05). The most enriched GO terms are shown in Figure 3D. The largest number of genes (n = 174) is assigned to the term of gene expression, which demonstrates the potential role of BADLNCR1 in regulation of gene expression.

### 3.4 GLRX5 is selected as target of BADLNCR1

**Figure 4:**
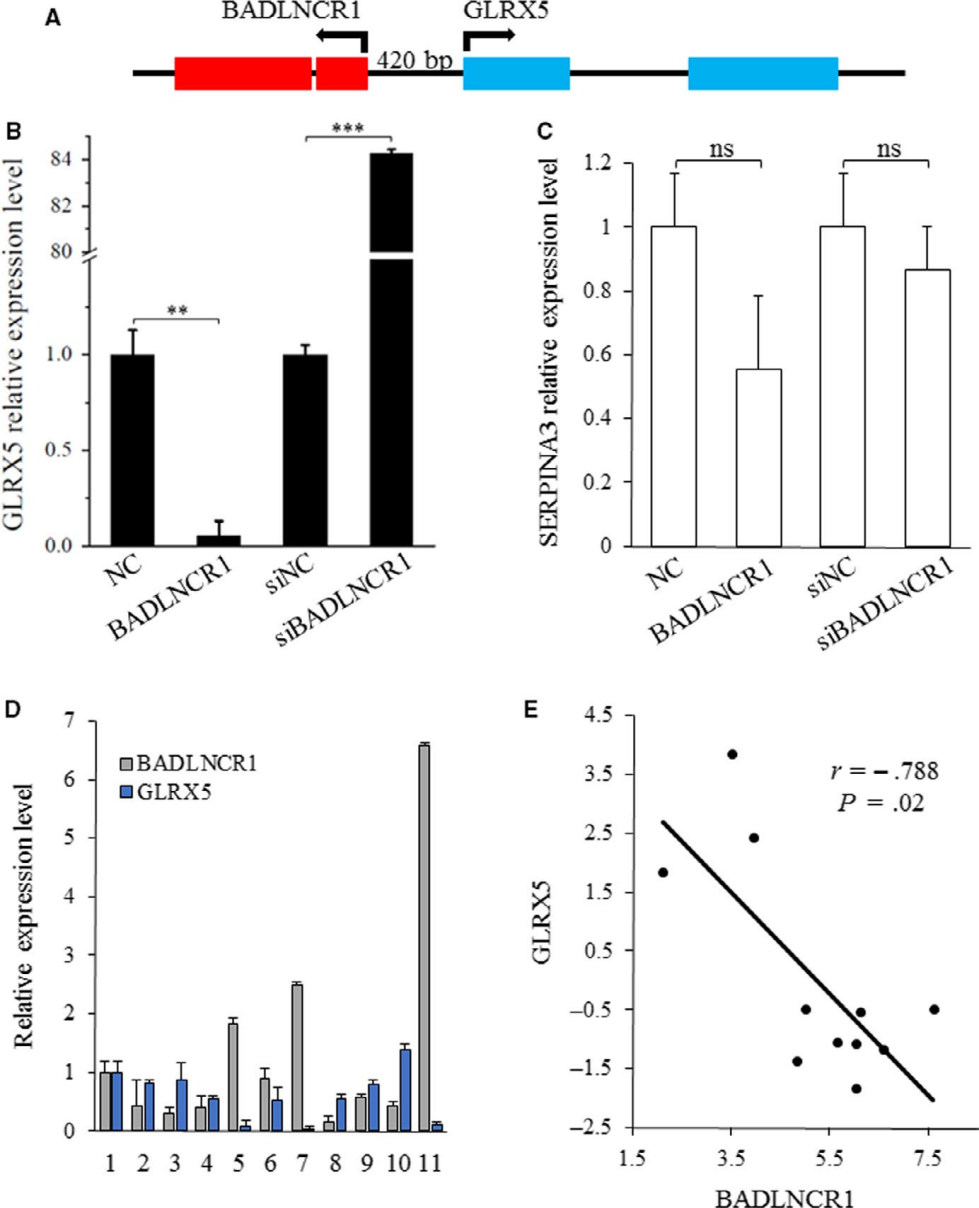
There is significantly negative correlation between the expression level of BADLNCR1 and GLRX5. A, Schematic illustration of the genomic location and structure of BADLNCR1 related to *GLRX5* gene. Red represents genomic location of BADLNCR1. Blue represents genomic location of *GLRX5*. B, There are significantly down‐regulated and up‐regulated mRNA expression of *GLRX5* gene after overexpression and knockdown of BADLNCR1, respectively. C, The expression levels of SERPINA3 do not change significantly either after overexpression of BADLNCR1 or BADLNCR1 knocked down. D, mRNA expression levels of BADLNCR1 and *GLRX5* in 11 adipose tissues of adult cattle. Their expression levels in sample 1 were defined as 1.0 for the comparisons. E, Correlation analysis between expression levels of BADLNCR1 and *GLRX5* gene. Data are presented as Mean ± SE. OV, overexpression. **P < .01, ***P < .001

To understand the molecular basis of how BADLNCR1 regulates adipocyte differentiation, using the University of California Santa Cruz (UCSC) Genome browser, transcripts that locate within 2000 kb of the BADLNCR1 locus were searched on chromosome 21, and those that overlap with the CHIRP‐seq data, *GLRX5* gene and serpin family A member 3 (SERPINA3) gene were selected for expression detection after overexpression or knocking down of BADLNCR1 in adipocytes. As a result, the mRNA expression level of *GLRX5* gene that locates 420 bp downstream of BADLNCR1 (Figure 4A) was significantly down‐regulated by BADLNCR1 overexpression (*P* < .05), as well as significantly up‐regulated by BADLNCR1 knocked down (*P* < .05) (Figure 4B). And their direction of transcription is reverse. BADLNCR1 could not change the expression of SERPINA3 gene significantly (*P* > .05) (Figure 4C). In addition, the expression levels of *GLRX5* gene and BADLNCR1 in 11 adult adipose tissues were evaluated (Figure 4D). Consistently, correlation analysis indicated that there was a significant negative correlation (*P* < .05, *r* < 0) between the expression levels of them (Figure 4E). Therefore, *GLRX5* gene was selected as the *cis*‐regulation of molecular target by BADLNCR1.

### 3.5 GLRX5 promotes bovine adipogenic differentiation

**Figure 5:**
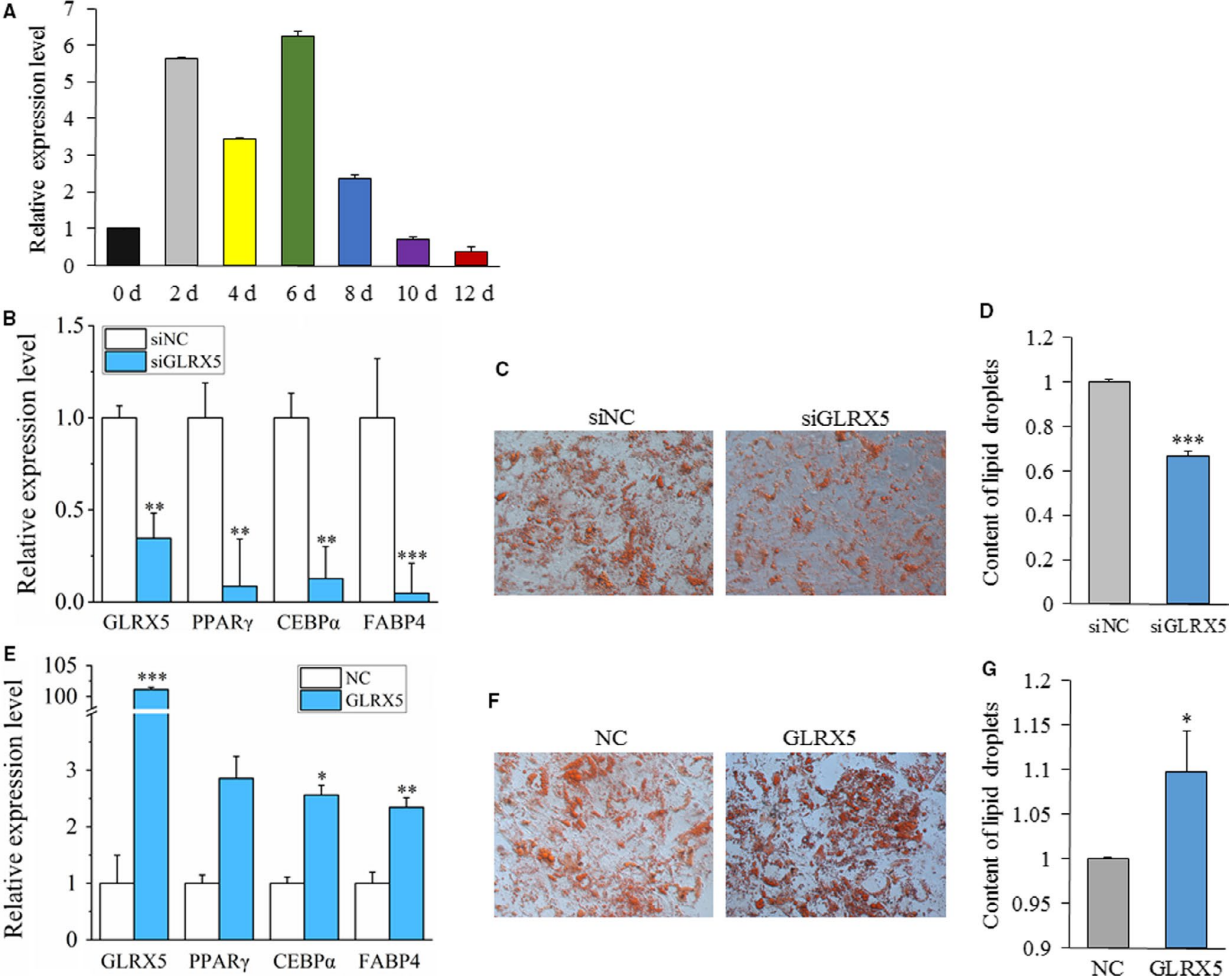
*GLRX5* promotes bovine adipogenic differentiation. A, Expression dynamics of *GLRX5* during adipocyte differentiation. B, Knock‐down of *GLRX5* suppresses mRNA expression of PPARγ, CEBPα and FABP4. C, Red Oil staining of lipid droplets after *GLRX5* knocked down. D, Content of lipid droplets calculated by ImageJ after *GLRX5* knocked down. E, Overexpression of *GLRX5* promotes mRNA expression of PPARγ, CEBPα, and FABP4. F, Red Oil staining of lipid droplets after overexpression of *GLRX5*. G, Content of lipid droplets calculated by ImageJ after overexpression of *GLRX5*. Data are presented as Mean ± SE. *P < .05, **P < .01, ***P < .001

Next, the involvement of *GLRX5* in bovine adipogenic differentiation was tested. *GLRX5* gene shows a rising expression trend after adipogenic induction (Figure 5A), which is opposite to the decreasing expression trend of BADLNCR1. After GLRX5 was knocked down by siRNA and siGLRX5, expression levels of PPARγ, CEBPα and FABP4 were significantly reduced (*P* < .05) (Figure 5B). Accordingly, significantly reducing amount of lipid droplets was observed by Red Oil Staining (Figure 5C,D). On the contrary, after the overexpression of *GLRX5*, the expression levels of CEBPα and FABP4 significantly increased (*P* < .05) and almost a twice rising of PPARγ was observed (*P* = .056) (Figure 5E). Also, more lipid droplets were formed (Figure 5F,G). Collectively, *GLRX5* gene is a positive regulator of bovine adipocyte differentiation. The effects of GLRX5 and BADLNCR1 are opposite.

### 3.6 BADLNCR1 represses GLRX5 transcription activity

Since BADLNCR1 regulates the mRNA expression of *GLRX5*, and it is transcribed from the upstream promoter region of *GLRX5*, it was supposed that BADLNCR1 was related to the transcription of *GLRX5*. Based on previous reports, there are several ways in which lncRNAs could regulate transcription. For example, lncRNAs function through binding to histone‐modifying complexes, to DNA‐binding proteins (including transcription factors) and even to RNA polymerase II.^41^ In addition, RNA can inhibit the binding of a transcriptional regulatory factor by acting as a “decoy” or inhibit its activity by direct active‐site occlusion.^42^ As a result, two hypotheses were posed that BADLNCR1 could bind to DNA‐binding protein or inhibit the binding of transcriptional factors and then suppress the transcription of *GLRX5*.

In order to test the former hypothesis, RNA pull‐down assay and mass spectrum were performed. Sixty‐two proteins were enriched. The top 10 that highly expressed with ≥99% matching are osteoglycin, tropomyosin 1, vimentin, asporin, lumican, troponin T3, proline and arginine‐rich end leucine‐rich repeat protein, collagen type VI alpha 2 chain, dermatopontin, and caveolae‐associated protein 1. However, there were no proteins related to histone‐modifying complexes, transcriptional factors or RNA polymerase II found. As a result, BADLNCR1 cannot recruit a regulatory protein to regulate the transcription of *GLRX5*.

**Figure 6:**
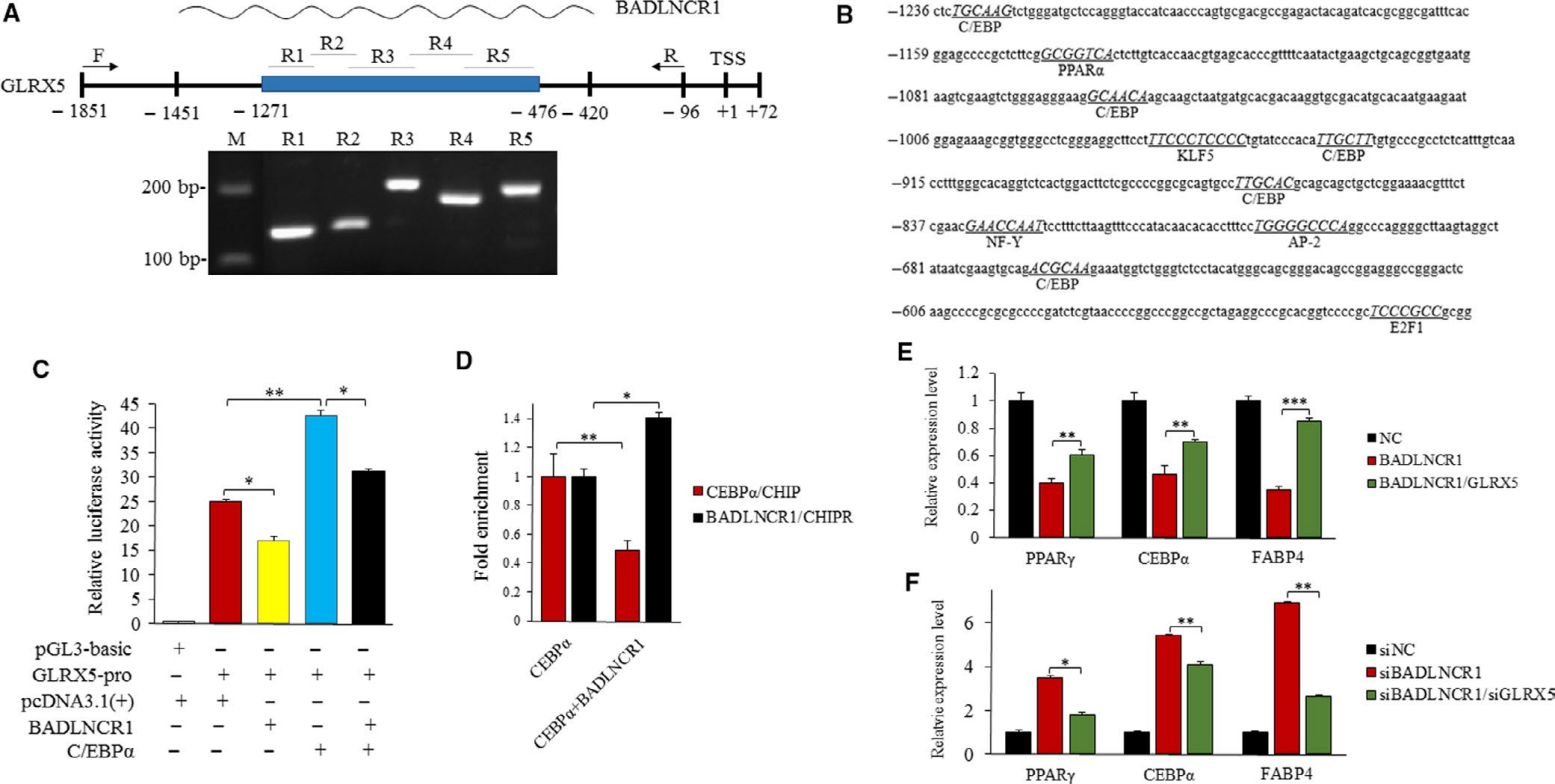
BADLNCR1 regulates transcription of *GLRX5*. A, Upper: schematic diagram of the binding site of BADLNCR1 on GLRX5 promotor, nether: result of CHIRP‐PCR. DNA marker is Marker I. B, Analysis of the putative adipogenic transcription factors that bind on the promotor of GLRX5. The consensus sequence for putative binding sites of transcriptional factors is underlined, in capital and italic. C, Individual or joint effects of BADLNCR1 and/or CEBPα on transcriptional activity of GLRX5 promoter detected by dual‐luciferase detection system. D, CHIP‐PCR and CHIRP‐PCR were used to detect the enrichment of CEBPα and BADLNCR1 on GLRX5 promoter after the overexpression of BADLNCR1. E, Overexpression of GLRX5 rescued the prevention of overexpression of BADLNCR1 on adipogenic gene expression. F, siGLRX5 reduced the enhancement of siBADLNCR1 on adipogenic gene expression. Data are presented as Mean ± SE. *P < .05, **P < .01, ***P < .001

To test the second hypothesis, CHIRP‐PCR was applied to verify the binding sites of BADLNCR1 on *GLRX5*. According to the CHIRP‐seq data, BADLNCR1 was found to bind on the upstream region (from −1271 to −476 bp) of *GLRX5* gene (Figure 6A). Based on the sequence of binding region, 5 pairs of primers (Table S2) were designed to perform PCR with DNA products from CHIRP assay as template to verify the sequencing data (Figure 6A). The lengths of amplified fragments were the same as expect. And the sequencing results of these fragments are the same as reference sequence. So BADLNCR1 binds on −1271 to −476 bp region of *GLRX5* gene. Bioinformatics analysis of this binding region revealed 5 binding sites for adipogenic transcriptional factors including CEBPα, activator protein 2, PPARα, KLF5, E2F transcription factor 1 and nuclear transcription factor Y (Figure 6B). As CEBPα is the most significantly predicted and its number of binding sites was the most, it was taken as an example for following assay. −1851 to −96 bp upstream region of *GLRX5* gene was constructed into pGL3 vector to be the reporter. As shown in Figure 6C, dual‐luciferase activity assay results indicated that overexpression of BADLNCR1 significantly reduces the relative luciferase activity of *GLRX5* promoter (*P* < .05), while overexpression of CEBPα significantly rises the relative luciferase activity of *GLRX5* promoter (*P* < .01). However, BADLNCR1 also significantly eliminates the enhancement of CEBPα on *GLRX5* promoter (*P* < .05). On the other hand, CHIP‐PCR and CHIRP‐PCR were performed using chromatins from NC or BADLNCR1 overexpressing bovine adipocytes to detect capacity of BADLNCR1 and CEBPα on *GLRX5* promoter. As expected, BADLNCR1 overexpression significantly reduced CEBPα enrichment at GLRX5 promoter along with significantly increasing enrichment of BADLNCR1 at GLRX5 promoter (Figure 6D). Taken together, these results indicated that BADLNCR1 may prevent the binding and activation of CEBPα to *GLRX5* promoter, ultimately resulting in obstruction to bovine adipogenic differentiation.

### 3.7 GLRX5 rescues inhibitory role of BALDNCR1 in bovine adipogenic differentiation

As *GLRX5* was selected as the target gene of BADLNCR1, and it plays opposite part to BADLNCR1 in bovine adipogenic differentiation, whether *GLRX5* could rescue the inhibitory role of BADLNCR1 was tested. As mentioned above, in bovine adipocyte, overexpression of BADLNCR1 inhibits the expression of PPARγ, CEBPα and FABP4. However, if *GLRX5* was overexpressed at the same time, their repressed expression levels were increased significantly (*P* < .01) (Figure 6E). Additionally, when BADLNCR1 was knocked down, these adipogenic genes displayed decreasing expression. But these decreasing levels were significantly increased after *GLRX5* was knocked down in the meantime (*P* < .01) (Figure 6F). Taken together, *GLRX5* may help to rescue the prevention of BADLNCR1 in bovine adipocyte differentiation.

## 4 DISCUSSION

Recently, more and more studies highlighted the importance of lncRNA in diverse biological processes, including regulating cell differentiation. Current research hot spots of lncRNA are mainly focused on tumorigenesis and stem cell pluripotency, while little is known on its role in adipogenesis, especially regarding their regulation of bovine adipogenic differentiation. Because of the treatment of metabolic disease in medicine science^43^, ^44^ and improvement of meat quality in livestock science,^45^, ^46^, ^47^ it is necessary to study molecular mechanisms underlying adipogenesis. lncRNAs have become as a novel class of regulator molecules, like protein regulators, that take an active part in multiple biological processes. Using RNA‐seq, Sun et al identified 175 lncRNAs that are specifically regulated during adipogenesis.^14^ However, how they regulate adipogenesis does not reveal. Using Ribo‐Zero RNA‐seq, Li et al found a stringent set of 2882 lncRNAs, and the regulatory mechanism of a competitive endogenous lncRNA ADNCR was identified.^15^ Based on Li's database, present research identified a nuclear lncRNA BADLNCR1 as a repressor of bovine adipocyte differentiation and its role in transcription regulatory was revealed. This study enriched the knowledge about molecular mechanisms underlying bovine adipogenesis.

The expression of BADLNCR1 is significantly reduced during bovine adipocyte differentiation, but not on the 2nd day post‐induction of adipogenesis. Adipocyte differentiation can be divided into two steps, proliferation and mitotic colony expansion (first step) and terminal differentiation (last step).^2^ Therefore, according to the expression trend of BADLNCR1 during adipogenesis, BADLNCR1 was supposed to be associated with adipogenic terminal differentiation. The following cellular experiment performed on 6th day showed that BADLNCR1 inhibits terminal adipocyte differentiation, which is consistent with its decreasing expression in terminal differentiation stage. No change in expression was found on 2nd day may indicate no effect of BADLNCR1 on pre‐adipocyte proliferation. However, further gain‐ and loss‐of‐function experiments are needed to explore whether BADLNCR1 regulates pre‐adipocyte proliferation or not.

As a member of GLRX family, a group of oxidoreductases catalyse the reversible reduction in protein disulphides^48^ and were involved in the biogenesis of iron‐sulphur clusters, *GLRX5* was identified as the downstream target of BADLNCR1. Using gain‐ and loss‐of‐function experiments, *GLRX5* was found to perform as a simulative gene that regulates bovine adipocyte differentiation. Iron‐sulphur cluster is necessary for the activity of a great number of mitochondrial proteins that participate in oxidative pathways, including complexes I and II of the respiratory chain.^49^, ^50^ Previous reports explained that oxidative pathways are associated with adipogenesis.^51^ And several iron‐sulphur cluster–related proteins were identified to be involved in adipose development, such as CISD1^52^ and MitoNEET.^53^ Also, *GLRX5* would be a gene candidate for the classification and brown adipocyte proportion estimation of brown adipose tissue and white adipose tissue.^54^ Taken this study together, it is enough to prove that *GLRX5* take a role in adipogenesis.

CHIRP assay showed that there is a binding site of BADLNCR1 on *GLRX5* promoter. BADLNCR1 is primarily localized in the nucleus, and BADLNCR1 negatively regulates the mRNA expression of *GLRX5*, so the association of BADLNCR1 with *GLRX5* looks at the regulation of BADLNCR1 on *GLRX5* promoter transcriptional activity. As expected, report gene study indicated that BADLNCR1 suppresses the transcriptional activity of *GLRX5* promoter, which is agreed with the negative correlation between the expression of BADLNCR1 and *GLRX5* and the opposite function of them two in regulating adipocyte differentiation. There are several ways in which lncRNAs could regulate gene transcription. First, lncRNA can recruit a regulatory protein to a gene, such as B2 RNA, which regulates RNA polymerase II by direct binding.^55^ Secondly, they can inhibit the binding of DNA‐binding protein, like Kcnq1ot1, Air and ROR.^56^, ^57^, ^58^ Also, transcription of lncRNA can influence the transcription of nearby genes.^59^ Finally, lncRNA can organize some domains of chromatin and then control these domains.^60^ Using RNA pull‐down assay, no physical interaction between BADLNCR1 and transcription related protein was observed, so it supposed that BADLNCR1 cannot recruit regulatory protein to regulate *GLRX5* transcription. As adipogenic transcriptional factors were predicted to bind on the promoter of *GLRX5*, like CEBPα, it supposed that BADLNCR1 impairs the enhancement of these transcriptional factors on *GLRX5* promoter activity. CHIP‐PCR demonstrated that overexpression of BADLNCR1 significantly reduces the enrichment of CEBPα on *GLRX5* promoter, which indicated that BADLNCR1 impairs the binding of CEBPα on *GLRX5* promoter.

Conservation is not a general feature for lncRNAs, especially in their nucleotide sequences. However, recent studies have indicated that lncRNAs show cross‐species conservation of their genomic position.^61^ For example, both mouse linc‐YY1 and human linc‐YY1 are transcribed from upstream genomic region of YY1 gene.^12^,^26^ Both mouse lncMyoD and human lncMyoD are generated from upstream of MyoD gene in both mouse and human genomes.^62^ Bioinformatics analysis indicated that mouse lncRNA Snhg10 and human lncRNA SNHG10 are reversely transcribed from upstream genomic region of GLRX5 gene (Figure S3A‐B). Consistently, BADLNCR1 is reversely transcribed from upstream genomic region of bovine GLRX5 gene. AnnoLnc web server shows that human lncRNA SNHG10 is detected in all tissues and highly expressed in fat (Figure S3C), and SNHG10 is mainly expressed in nuclear in most of cell lines as determined using the lncATLAS annotation database (Figure S3D), these patterns similar to those of bovine BADLNCR1. However, expression levels of mouse Snhg10 in pre‐adipocytes and mature adipocytes have no significant difference in 3T3‐L1 (Figure S3E). Considering the important role of BADLNCR1 in bovine adipogenesis, further researches of the function and mechanisms of SHNG10 in human adipogenesis and mouse adipogenesis should be performed.

**Figure 7:**
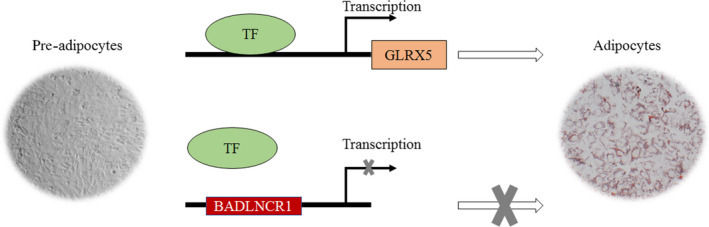
Model depicting how BADLNCR1 inhibits bovine adipocyte differentiation. TF, transcriptional factor

In a conclusion, *KLF2* regulated lncRNA, and BADLNCR1 appears to act as a suppressor during bovine adipogenic differentiation. This process might perform by its negative regulation on transcriptional activity and mRNA expression of *GLRX5*, which is benefit for bovine adipogenic differentiation (Figure 7). These findings support a mechanism underlying how lncRNA regulates bovine adipose development.

## CONFLICT OF INTEREST

The authors declare that they have no conflicts of interest with the contents of this article.

## AUTHOR CONTRIBUTIONS

HC designed and performed research and wrote paper; ML and JW performed experiments; CS analysed data; YH and XL collected samples; CL revised manuscript; HC conducted this research.

## Supporting information

Fig S1‐S3

Table S1‐S5

## Data Availability

The data sets generated or analysed during the current study are available from the first authors and corresponding author on reasonable request.
