# Supplementary material for: A novel lncRNA BADLNCR1 inhibits bovine adipogenesis by repressing GLRX5 expression
Source: J Cell Mol Med. 2020 May 25;24(13):7175–86. doi: 10.1111/jcmm.15181 (PMC7339203; doi:10.1111/jcmm.15181)
Supplement: Supplementary file 1 — Fig S1‐S3 [file JCMM-24-7175-s001.docx]

**
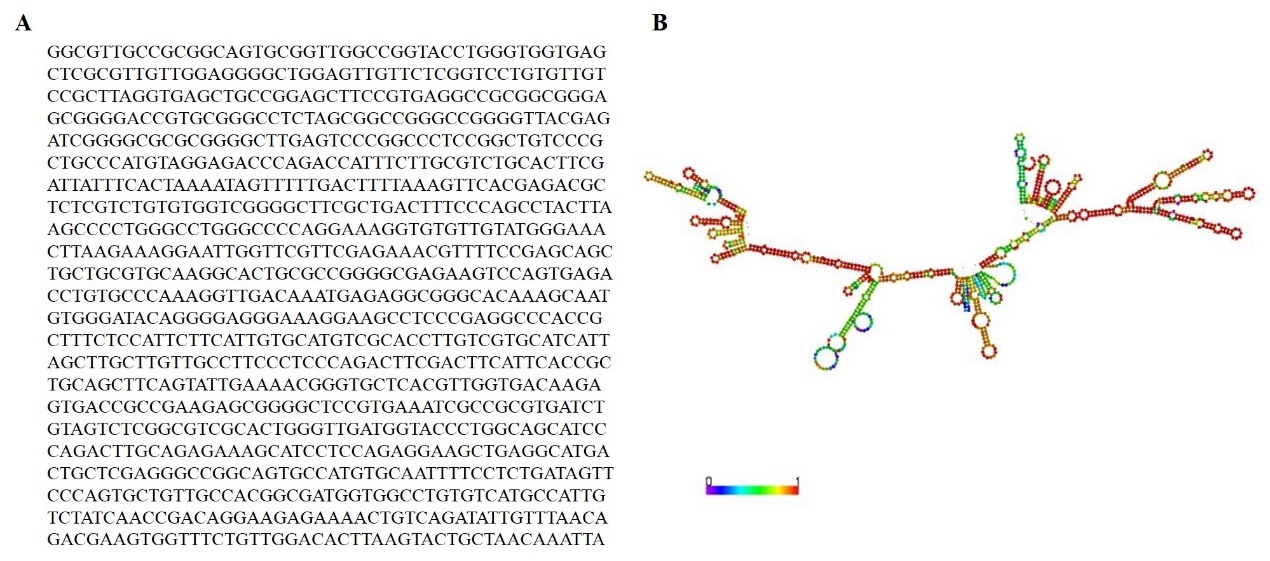
**

**Figure S1** Sequence characteristics of BADLNCR1. (A) Full nucleotide sequence of BADLNCR1. (B) Secondary structure of BADLNCR1 predicted by RNAfold.


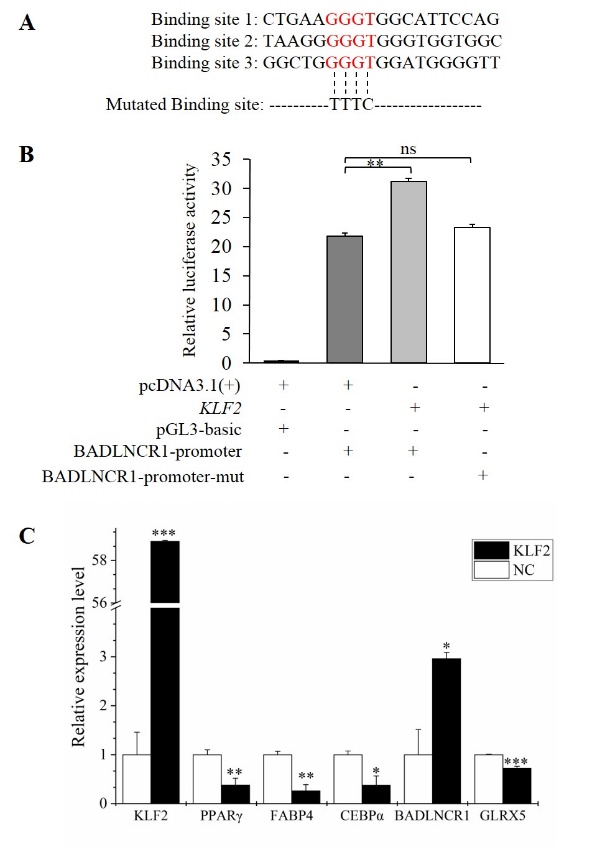


**Figure S2** KLF2 regulates transcriptional activity and expression of BADLNCR1. (A) Binding sites of KLF2 on BADLNCR1 promoter. “GGGT” was replaced by “TTTC” in mutated report gene vector. (B) KLF2 represses the relative luciferase activity of BADLNCR1 promoter. (C) Effects of KLF2 on expression of adipogenic genes, BADLNCR1, and GLRX5. The data was represented as Mean±SE. **P*<0.05, ***P*<0.01, ****P*<0.001, ns indicates statistical non-significance.


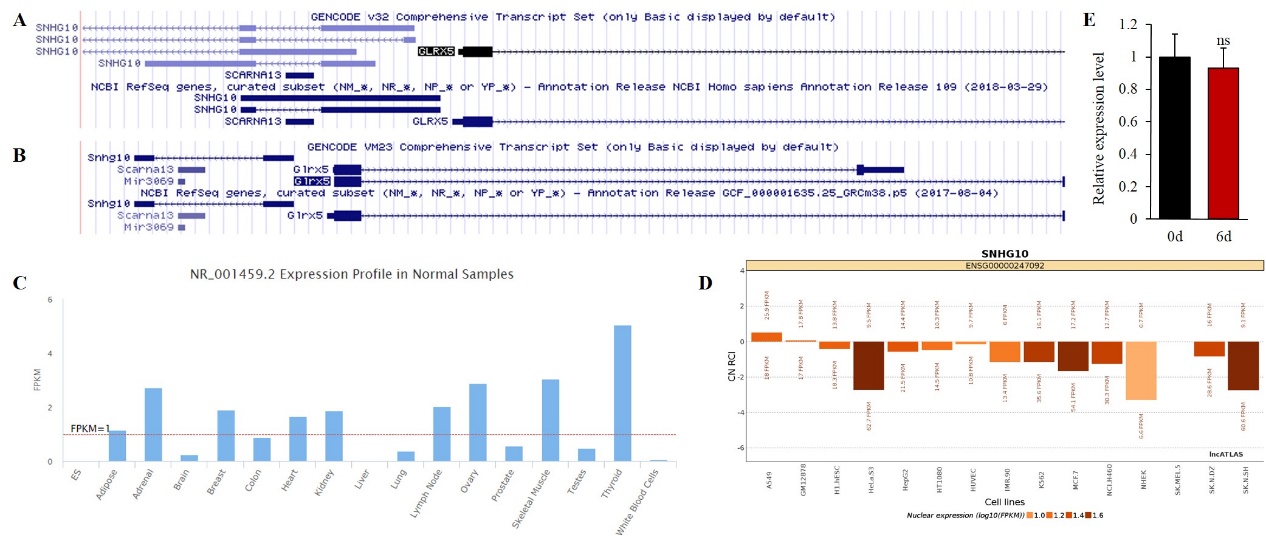


**Figure S3** Conservation analysis between BADLNCR1 and SNGH10/Sngh10 gene. (A) Localization of SNGH10 gene in human genome according to UCSC Genome Browser. (B) Localization of Sngh10 gene in mouse genome according to UCSC Genome Browser. (C) Tissue expression pattern of human SNGH10 gene in normal samples according to AnnoLnc database. (D) Human SNGH10 gene is mainly distributed in nuclei of multiple cell lines according to the AnnoLnc database. (E) Expression of Sngh10 gene in undifferented (0d) and differentiated (6d) 3T3-L1. The data was represented as Mean±SE. ns indicates statistical non-significance.
